# Double and Square Bessel–Gaussian Beams

**DOI:** 10.3390/mi14051029

**Published:** 2023-05-11

**Authors:** Eugeny G. Abramochkin, Victor V. Kotlyar, Alexey A. Kovalev

**Affiliations:** 1 Lebedev Physical Institute, Novo-Sadovaya 221, 443034 Samara, Russia; 2 Image Processing Systems Institute of the RAS—Branch of FSRC “Crystallography & Photonics” of the RAS, 151 Molodogvardeyskaya St., 443001 Samara, Russia; 3 Samara National Research University, 34 Moskovskoe Shosse, 443086 Samara, Russia

**Keywords:** Bessel–Gaussian beam, optical vortex, paraxial propagation

## Abstract

We obtain a transform that relates the standard Bessel–Gaussian (BG) beams with BG beams described by the Bessel function of a half-integer order and quadratic radial dependence in the argument. We also study square vortex BG beams, described by the square of the Bessel function, and the products of two vortex BG beams (double-BG beams), described by a product of two different integer-order Bessel functions. To describe the propagation of these beams in free space, we derive expressions as series of products of three Bessel functions. In addition, a vortex-free power-function BG beam of the *m*th order is obtained, which upon propagation in free space becomes a finite superposition of similar vortex-free power-function BG beams of the orders from 0 to *m*. Extending the set of finite-energy vortex beams with an orbital angular momentum is useful in searching for stable light beams for probing the turbulent atmosphere and for wireless optical communications. Such beams can be used in micromachines for controlling the movements of particles simultaneously along several light rings.

## 1. Introduction

Among different light beams, there are not many beam families whose propagation in free space is described analytically. Thus, to predict beams’ behavior upon propagation, focusing, or other conversions, it is often necessary to use some numerical approaches which can be inexact and cannot explain the physical effects that can appear or disappear when some parameters are changed. Thus, finding a new light beam that can be described analytically is a significant achievement. Among the paraxial vortex light beams that have an analytical description, the most widely known are the Laguerre–Gaussian [1] or Bessel–Gaussian beams [2]. Other examples of such beams are hypergeometric Gaussian beams [3] and modes [4] or generalized Laguerre–Gaussian beams [5].

Starting from the well-known work by F. Gori and co-authors [2], miscellaneous modifications of the Bessel–Gaussian (BG) beams are being investigated with a growing interest. The BG beams can be generated by an axicon, a light modulator, or other elements [6,7,8]. Bessel–Gaussian beams are convenient for probing the turbulent atmosphere since, on one hand, they are of a finite energy like the Gaussian beam and, on the other hand, they manifest quasi-diffraction-free properties due to the Bessel function [9,10,11,12]. As was shown in [13], BG beams are more resistant to the distortions induced by a turbulent atmosphere than the Gaussian beam. The authors of [14] demonstrated that in a Kolmogorov-type turbulent atmosphere, BG beams conserve along a longer distance than the Gaussian beam. The free-space propagation of BG beams in the THz wavelength range was studied in [15]. Bessel–Gaussian beams are used for the manipulation of microparticles [16,17] and for generating entangled pairs of photons in quantum informatics [18,19]. Therefore, extending the set of different types and modifications of BG beams is a relevant problem. In [20], modified BG beams called “frozen waves” were studied. In [21], the propagation of asymmetric BG beams [22,23] in a turbulent atmosphere was considered.

In this work, we obtain modifications of BG beams, such as square BG beams and products of the BG beams. For such beams, we derive explicit analytical expressions that describe their evolution upon propagation in free space. Similar to standard BG beams, the beams we study are not structurally stable, but they are also composed of a set of concentric light rings. The number of rings and the light energy between them change upon the beam’s propagation in free space. In the far field, all the beams studied except one are similar to the standard BG beam and contain a single light ring with almost no side lobes. Only one beam, the vortex-free power-function BG beam, contains several rings in the far field, and their number equals that of the beam order.

## 2. Bessel–Gaussian Beams and Modulated Bessel–Gaussian Beams

Bessel–Gaussian (BG) beams, first studied in [2], are not structurally stable beams, i.e., their transverse intensity distribution changes when the beams propagate in free space. However, the change is insignificant, and all the intensities keep their shape of concentric circles. The complex amplitude of the BG beam at an arbitrary distance along the optical axis *z* is

(1) $${\mathrm{BG}}_{m}(r,\varphi ,z\left|c\right.)=\frac{1}{q(z)}\exp\left(\frac{c^{2}}{4q(z)}-\frac{r^{2}}{w_{0}^{2}q(z)}+im\varphi \right)J_{m}\left(\frac{cr}{w_{0}q(z)}\right),$$

where $q(z)=1+iz/z_{0}$, $z_{0}=kw_{0}^{2}/2$ is the Rayleigh range, (*r*, *φ*) are the polar coordinates in the transverse plane, *m* is the topological charge, *J_m_*(*x*) is the *m*th-order Bessel function of the first kind, *w*_0_ is the Gaussian beam waist radius, *k* is the wavenumber of light, *c* is a dimensionless (possibly complex) parameter affecting the transverse component of the wave vector (*c*/*w*_0_ = *k_r_*), and $k^{2}=k_{r}^{2}+k_{z}^{2}$, *k_z_* is the longitudinal component of the wave vector. Here and in the following text, we will use a vertical line to separate variables and parameters.

The complex amplitude of a BG beam (1) in the initial plane can be expanded into a series of Laguerre–Gaussian (LG) beams:

(2) $${\mathrm{BG}}_{m}(r,\varphi ,0\left|c\right.)=\exp\left(\frac{c^{2}}{4}-\frac{r^{2}}{w_{0}^{2}}+im\varphi \right)J_{m}\left(\frac{cr}{w_{0}}\right)=\exp\left(\frac{c^{2}}{8}\right)\sum_{\nu =0}^{\infty }\frac{c^{2\nu +m}{\mathrm{LG}}_{\nu ,m}(r,\varphi )}{2^{3\nu +m}(\nu +m)!},$$

where the LG beams in the initial plane are described by the following expression:

(3) $${\mathrm{LG}}_{n,m}(r,\varphi )=\exp\left(-\frac{r^{2}}{w_{0}^{2}}+im\varphi \right){\left(\frac{r}{w_{0}}\right)}^{m}L_{n}^{m}\left(\frac{2r^{2}}{w_{0}^{2}}\right),$$

Based on the associated Laguerre polynomial $L_{n}^{m}(x)$. As can be seen from Equation (2), when the parameter *c* is small (c≪1), only the first term in the series is significant, and the BG beam in this case is close to the LG beam.

In addition to standard BG beams, BG beams with quadratic radial dependence (qBG) are also known. They were introduced in [24]. Later, in [25], the transformation of these beams by an optical ABCD system was investigated, and now qBG beams are considered part of the classification of circular optical beams [26]. In the initial plane, the complex amplitude of a qBG beam is defined as

(4) $${\mathrm{qBG}}_{m}(r,\varphi ,0\left|c\right.)=\exp\left(-\frac{r^{2}}{w_{0}^{2}}+im\varphi \right)J_{m/2}\left(\frac{cr^{2}}{w_{0}^{2}}\right).$$

As can be seen from Equation (4), the qBG beams depend on a Bessel function of an integer order when *m* is even and a Bessel function of a half-integer order when *m* is odd. It can also be seen that in contrast with BG beams, the argument of the Bessel function depends quadratically on the radial coordinate. The complex amplitude of a qBG beam in the Fresnel zone is as follows:

(5) $${\mathrm{qBG}}_{m}(r,\varphi ,z\left|c\right.)=\frac{1}{\sqrt{q_{+}q_{-}}}\exp\left(-\left[1+i(1+c^{2})\frac{z}{z_{0}}\right]\frac{r^{2}}{w_{0}^{2}q_{+}q_{-}}+im\varphi \right)J_{m/2}\left(\frac{cr^{2}}{w_{0}^{2}q_{+}q_{-}}\right).$$

The parameters *q*_±_ in Equation (4) depend on the propagation distance *z* and the parameter *c*:

(6) $$q_{\pm }=q_{\pm }(z)=q(z)\pm c\frac{z}{z_{0}}=1+\left(i\pm c\right)\frac{z}{z_{0}}.$$

Equations (5) and (6) reveal that when the value *c* is large enough (c≫1), the imaginary part of the factor *q*_+_*q*_−_ in the argument of the Bessel and the exponential functions is small compared to the real part, and the BG becomes approximately propagation-invariant, i.e., its intensity distribution shape is almost conserved, changing only in scale.

Further, we obtain an integral transform that relates BG beams (1) with qBG beams (4). It can be shown that this transform is given by

(7) $$\begin{array}{c} F(r,\varphi ,z\left|a\right.)=\int_{0}^{\infty }{\mathrm{BG}}_{m}\left(r,\varphi ,z\left|c\right.\right)J_{m/2}\left(\frac{ac^{2}}{4}\right)cdc\\ \;=\frac{2}{\sqrt{1+a^{2}q^{2}(z)}}\exp\left(-\frac{a^{2}q(z)r^{2}}{1+a^{2}q^{2}(z)}+im\varphi \right)J_{m/2}\left(\frac{ar^{2}}{1+a^{2}q^{2}(z)}\right). \end{array}$$

Since

$$F\left(r,\varphi ,z+i\cdot \frac{c^{2}}{1+c^{2}}\left|\frac{1+c^{2}}{c}\right.\right)={\mathrm{qBG}}_{m}(r,\varphi ,z\left|c\right.),$$

the complex source idea then reveals a common background of both beams, described in Equations (1) and (5).

## 3. Square Bessel–Gaussian Beams

In our works [27,28], we considered double Laguerre–Gaussian (dLG) beams and square LG beams. Both of these types of beams can be expressed via finite sums of LG beams. In [29], (Chapter X “Orthogonal polynomials”, Section 10.12 “Laguerre polynomials”), the following relation between the Laguerre polynomials and the Bessel function is given:

(8) $$\underset{n\to \infty }{\lim}\left[n^{-\alpha }L_{n}^{\alpha }\left(r^{2}/n\right)\right]=r^{-\alpha }J_{\alpha }\left(2r\right).$$

Thus, it can be seen that when the radial index tends to infinity, the number of light rings should also tend to infinity, and the radii of these rings are described by the Bessel function. This leads to the qualitative difference between the Laguerre–Gaussian and the Bessel–Gaussian beams—the former have a finite number of rings, and the latter have an infinite number of rings. A natural continuation of the works [27,28] is the investigation of whether similar solutions to the Helmholtz equation can be obtained that describe square BG beams or the products of the BG beams.

Here, we consider square BG beams and show that they can be represented as an infinite sum of BG beams (1). The complex amplitude of a square BG beam (BBG) in the initial plane reads as

(9) $${\mathrm{BBG}}_{m}(r,\varphi ,0\left|c\right.)=\exp\left(\frac{c^{2}}{2}-\frac{r^{2}}{w_{0}^{2}}+2im\varphi \right)J_{m}^{2}\left(\frac{cr}{w_{0}}\right).$$

Here, we prefer to scale the parameters of the initial BG beam ($c\to c\sqrt{2}$,$w_{0}\to w_{0}\sqrt{2}$) but keep the Gaussian factor unchanged.

The complex amplitude of the BBG beam can be obtained by using a generating function for the squares of the Bessel functions [30]:

(10) $$\sum_{\nu \in \mathbb{Z}}J_{\nu }^{2}(x){\left(-t^{2}\right)}^{\nu }=J_{0}\left[x\left(t+\frac{1}{t}\right)\right].$$

After rather complicated algebra, we reduce the Fresnel transform of the initial complex amplitude (9) to the following series of Bessel functions:

(11) $$\begin{array}{l} {\mathrm{BBG}}_{m}(r,\varphi ,z\left|c\right.)=\frac{1}{q(z)}\exp\left(\frac{c^{2}}{2q(z)}-\frac{r^{2}}{w_{0}^{2}q(z)}+2im\varphi \right)\\ \times \sum_{\nu \in \mathbb{Z}}{\left(-i\right)}^{\nu }J_{m-\nu }\left(\frac{cr}{w_{0}q(z)}\right)J_{m+\nu }\left(\frac{cr}{w_{0}q(z)}\right)J_{\nu }\left(\frac{c^{2}z}{2z_{0}q(z)}\right). \end{array}$$

Equation (11) indicates that the square BG beams do not conserve their shape upon propagation in free space but are a superposition of a countable number of the products of BG beams of the orders whose sum is equal to 2*m*, i.e., the initial topological charge. It is interesting to note that in the far field, i.e., when z→∞, the series in Equation (11) reduces to the power function *r*^2*m*^. Thus, in the far field (and in the focus of a spherical lens), the square BG beam has the shape of a single light ring without side lobes.

## 4. Product of Two Bessel–Gaussian Beams

Since the series in Equation (11) contains the product of two similar Bessel functions depending on *r*, it seems quite possible that for a product of two different BG beams, instead of the squared one, we can evaluate the Fresnel transform. Let us introduce the product of two BG beams in the initial plane *z* = 0:

(12) $${\mathrm{dBG}}_{m,n}(r,\varphi ,0\left|a,b\right.)=\exp\left(\frac{a^{2}+b^{2}}{4}-\frac{r^{2}}{w_{0}^{2}}+i(m+n)\varphi \right)J_{m}\left(\frac{ar}{w_{0}}\right)J_{n}\left(\frac{br}{w_{0}}\right).$$

Then, in same way as expansion (11) was derived, the initial field (12) leads to the following solution of the paraxial equation:

(13) $$\begin{array}{r} {\mathrm{dBG}}_{m,n}(r,\varphi ,z\left|a,b\right.)=\frac{1}{q(z)}\exp\left(\frac{a^{2}+b^{2}}{4q(z)}-\frac{r^{2}}{w_{0}^{2}q(z)}+i(m+n)\varphi \right)\\ \times \sum_{\nu \in \mathbb{Z}}{\left(-i\right)}^{\nu }J_{m+\nu }\left(\frac{ar}{w_{0}q(z)}\right)J_{n-\nu }\left(\frac{br}{w_{0}q(z)}\right)J_{\nu }\left(\frac{abz}{2z_{0}q(z)}\right). \end{array}$$

As can be seen from Equation (13), if n=m and *a* = *b* = *c*, the product of two BG beams reduces to the square BG beam. It can also be seen that for the case *n* = *b* = 0, the series in (13) collapses to the only term with ν=0, and the product of two BG beams reduces to the standard BG beam:

(14) $${\mathrm{dBG}}_{m,0}(r,\varphi ,z\left|a,0\right.)={\mathrm{BG}}_{m}(r,\varphi ,z\left|a\right.).$$

Some other particular cases of the dBG beams, as in Equation (13), are also interesting to mention. If n=−m, then the dBG beam is a vortex-free beam:

(15) $$\begin{array}{l} {\mathrm{dBG}}_{m,-m}(r,\varphi ,0\left|a,b\right.)={\left(-1\right)}^{m}\exp\left(\frac{a^{2}+b^{2}}{4}-\frac{r^{2}}{w_{0}^{2}}\right)J_{m}\left(\frac{ar}{w_{0}}\right)J_{m}\left(\frac{br}{w_{0}}\right),\\ {\mathrm{dBG}}_{m,-m}(r,\varphi ,z\left|a,b\right.)=\frac{{\left(-1\right)}^{m}}{q(z)}\exp\left(\frac{a^{2}+b^{2}}{4q(z)}-\frac{r^{2}}{w_{0}^{2}q(z)}\right)\\ \;\times \sum_{\nu \in \mathbb{Z}}i^{\nu }J_{m+\nu }\left(\frac{ar}{w_{0}q(z)}\right)J_{m+\nu }\left(\frac{br}{w_{0}q(z)}\right)J_{\nu }\left(\frac{abz}{2z_{0}q(z)}\right). \end{array}$$

Its limiting case, when *b* vanishes, is a product of the vortex-free BG beam by the power function:

(16) $$\begin{array}{c} {\mathrm{pBG}}_{m}(r,\varphi ,0\left|a\right.)=2^{m}m!\;\cdot \underset{b\to 0}{\lim}\frac{{\mathrm{dBG}}_{m,-m}(r,\varphi ,0\left|a,b\right.)}{{\left(-ab\right)}^{m}}\\ \;=\exp\left(\frac{a^{2}}{4}-\frac{r^{2}}{w_{0}^{2}}\right){\left(\frac{r}{aw_{0}}\right)}^{m}J_{m}\left(\frac{ar}{w_{0}}\right). \end{array}$$

The beam from Equation (16) can be called a vortex-free power-function BG beam. Tending the parameter *b* to zero in Equation (15), we obtain nonzero items of the series for −m≤ν≤0 only. Then, changing the summation index ν→−ν, we derive the Fresnel transform of the beam from Equation (16):

(17) $$\begin{array}{l} {\mathrm{pBG}}_{m}(r,\varphi ,z\left|a\right.)=\frac{1}{q(z)}\exp\left(\frac{a^{2}}{4q(z)}-\frac{r^{2}}{w_{0}^{2}q(z)}\right)\\ \times \sum_{\nu =0}^{m}\left(\begin{array}{c} m\\ \nu \end{array}\right){\left(\frac{r}{aw_{0}q(z)}\right)}^{m-\nu }J_{m-\nu }\left(\frac{ar}{w_{0}q(z)}\right){\left(\frac{iz}{z_{0}q(z)}\right)}^{\nu }. \end{array}$$

Although the square and products of the BG beams are given by infinite superpositions (Equations (11) and (13)), expression (17) indicates that the power-function vortex-free BG beam in the Fresnel diffraction zone is a finite superposition of similar power-function BG beams of the orders *ν* from 0 to *m*.

In the far field ($z\gg z_{0}$), the argument of the Bessel functions in Equation (17) becomes small. Thus, since $J_{\nu }(\xi )\approx {\left(\xi /2\right)}^{\nu }/\nu !$ at ξ≈0, the sum in Equation (17) transforms into an ordinary Laguerre polynomial:

(18) $$\begin{array}{l} {\mathrm{pBG}}_{m}(r,\varphi ,z\gg z_{0}\left|a\right.)\approx \frac{1}{q(z)}\exp\left(\frac{a^{2}}{4q(z)}-\frac{r^{2}}{w_{0}^{2}q(z)}\right)\sum_{\nu =0}^{m}\left(\begin{array}{c} m\\ \nu \end{array}\right)\frac{{\left(-z_{0}^{2}r^{2}/2z^{2}w_{0}^{2}\right)}^{m-\nu }}{(m-\nu )!}\\ \;=\frac{1}{q(z)}\exp\left(\frac{a^{2}}{4q(z)}-\frac{r^{2}}{w_{0}^{2}q(z)}\right)L_{m}\left(\frac{z_{0}^{2}r^{2}}{2z^{2}w_{0}^{2}}\right). \end{array}$$

As result, in the far field, the pBG beam appears as *m* light rings.

## 5. Simulation

In this section, we describe the computational results of the beams from Equations (5), (13) and (17). All distributions are obtained in two ways: by using the numerical Fresnel transform, implemented as a convolution with adopting the discrete fast Fourier transform, and using the theoretical expression. Using the discrete fast Fourier transform is actually equivalent to computing the convolution integrals via Rieman sums, i.e., splitting the integration area into rectangles. All intensity distributions obtained in these two ways are visually indistinguishable, while the phase distributions are different only in low-intensity areas. This confirms the correctness of the Formulae (5), (13) and (17) for the complex amplitudes upon space propagation.

**Figure 1:**
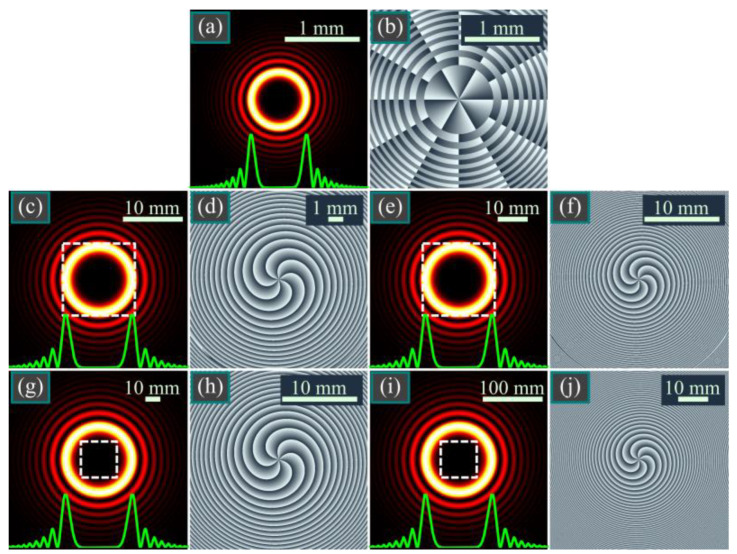
Intensity (**a**,**c**,**e**,**g**,**i**) and phase (**b**,**d**,**f**,**h**,**j**) distributions of the qBG beam (5) in several transverse planes for the following computation parameters: wavelength λ = 532 nm, Gaussian beam waist radius *w*_0_ = 1 mm, beam order *m* = 3, scaling factor *c* = 30, propagation distances *z* = 0 (**a**,**b**), *z* = *z*_0_/2 (**c**,**d**), *z* = *z*_0_ (**e**,**f**), *z* = 2*z*_0_ (**g**,**h**), and *z* = 5*z*_0_ (**i**,**j**). Dashed squares (**c**,**e**,**g**,**i**) denote the areas corresponding to shown phase distributions (**d**,**f**,**h**,**j**). Green plots (**a**,**c**,**e**,**g**,**i**) show the intensity cross-sections.

Shown in Figure 1 are the intensity and phase distributions of the modulated Bessel–Gaussian beam (5) in several transverse planes. To obtain a beam with several rings and to prove that it is approximately propagation-invariant, we choose a large value of the scaling factor *c* = 30.

Figure 1 confirms that when the scaling factor *c* is large enough, the transverse intensity shape almost does not change upon propagation.

**Figure 2:**
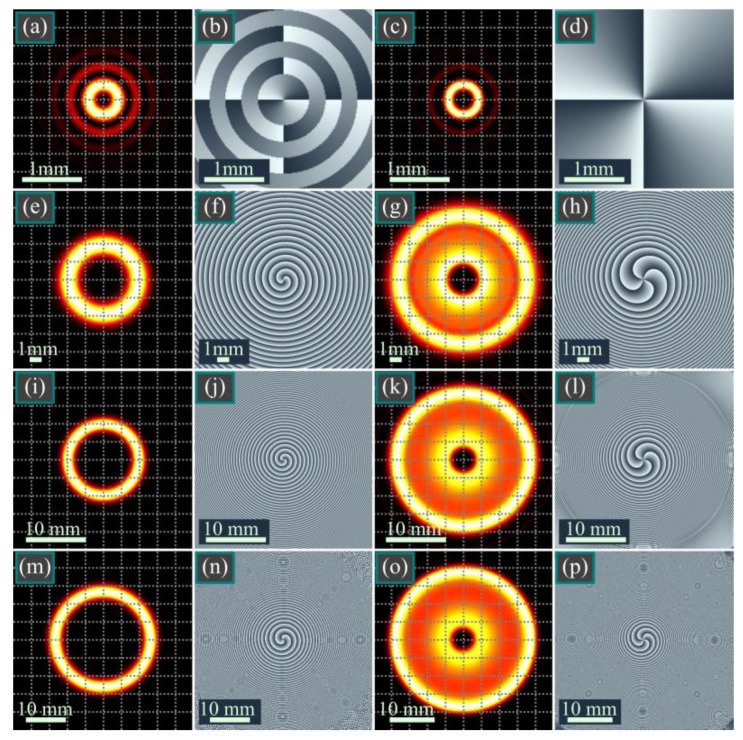
Intensity (**a**,**c**,**e**,**g**,**i**,**k**,**m**,**o**) and phase (**b**,**d**,**f**,**h**,**j**,**l**,**n**,**p**) distributions of a BG beam (**a**,**b**,**e**,**f**,**i**,**j**,**m**,**n**) and a square BG beam (**c**,**d**,**g**,**h**,**k**,**l**,**o**,**p**), Equations (9) and (11), in several transverse planes for the following computational parameters: wavelength λ = 532 nm, Gaussian beam waist radius *w*_0_ = 1 mm, beam orders *m* = *n* = 2, scaling factors *a* = *b* = 12, propagation distances *z* = 0 (**a**–**d**), *z* = *z*_0_/2 (**e**–**h**), *z* = *z*_0_ (**i**–**l**), and *z* = 2*z*_0_ (**m**–**p**).

Figure 2 depicts the intensity and phase distributions of a BG beam and of the corresponding square BG beam (9) (11) in several transverse planes.

According to Figure 2, the square BG beam is narrower in the initial plane, and its side lobes are suppressed due to the squared Bessel function. A narrower distribution in the initial plane leads to higher space frequencies and thus to higher divergence upon propagation in free space. This is confirmed by Figure 2g,k,o. A notable feature of the squared BG beams is the much smaller dark area in the center of the diffraction pattern.

**Figure 3:**
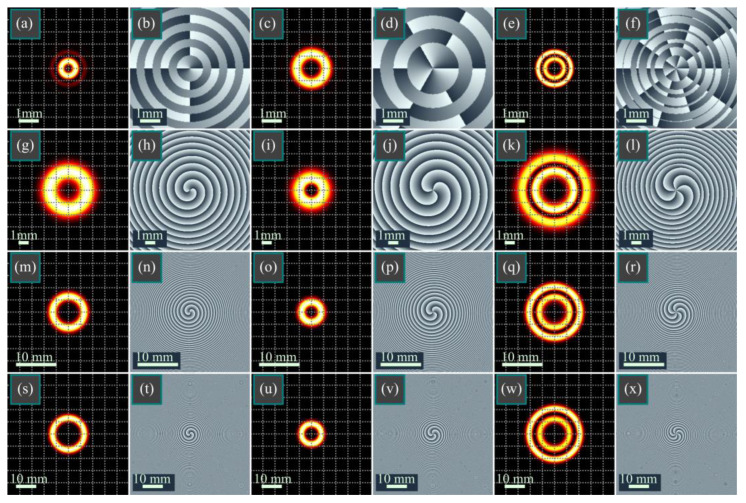
Intensity (**a**,**c**,**e**,**g**,**i**,**k**,**m**,**o**,**q**,**s**,**u**,**w**) and phase (**b**,**d**,**f**,**h**,**j**,**l**,**n**,**p**,**r**,**t**,**v**,**x**) distributions of two BG beams with different parameters (**a**–**d**,**g**–**j**,**m**–**p**,**s**–**v**), as well as of the beams described in Equations (12) and (13), constructed as their product (**e**,**f**,**k**,**l**,**q**,**r**,**w**,**x**), in several transverse planes for the following computational parameters: wavelength λ = 532 nm, Gaussian beam waist radius *w*_0_ = 1 mm, orders of BG beams *m* = 2 (**a**,**b**,**g**,**h**,**m**,**n**,**s**,**t**) and *n* = 3 (**c**,**d**,**i**,**j**,**o**,**p**,**u**,**v**), scaling factors of BG beams *a* = 8 (**a**,**b**,**g**,**h**,**m**,**n**,**s**,**t**) and *b* = 5 (**c**,**d**,**i**,**j**,**o**,**p**,**u**,**v**), propagation distances *z* = 0 (**a**–**f**), *z* = *z*_0_/2 (**g**–**l**), *z* = *z*_0_ (**m**–**r**), and *z* = 2*z*_0_ (**s**–**x**). The grids on the intensity distributions are shown to match the radii of the light rings in different beams.

Figure 3 illustrates the intensity and phase distributions of two BG beams with different parameters, as well as of the beams described in (12) and (13), constructed as their product, in several transverse planes. For computation based on Equation (13), the series was bounded by 100 terms.

As can be seen in Figure 3, the intensity distributions of both BG beams in the initial plane have the shape of a single bright ring (Figure 3a,c), but the first beam has a ring with a smaller radius and a pale second ring (Figure 3a). Between these rings, there is a dark, zero-intensity ring, and the multiplication of the complex amplitudes of both beams (two multipliers in Equation (12)) leads to two bright, light rings (Figure 3e), since the thick ring in Figure 3c is “cut” into two rings by the dark ring from Figure 3a. This is a key difference here from the square BG beams, i.e., instead of one dark spot in the center of a circular light distribution, the diffraction pattern contains a dark ring between two light rings.

In the initial phase distributions (Figure 3b,d,f), there are rings with phase jumps of π. The Bessel functions are equal to zero on these rings. However, upon propagation, the arguments of the Bessel functions become complex, and the function values are nonzero. Therefore, there are no such phase jumps in Figure 3h,j,l,n,p,r,t,v,x.

Despite the lower topological charge of the first BG beam (*m* = 2 vs. *n* = 3), its scaling factor is, vice versa, greater than that of the second beam (*a* = 8 vs. *b* = 5). Therefore, upon propagation, it diverges faster, and at a distance of *z* = *z*_0_/2, its light ring has a greater diameter than the ring of the second BG beam; it is now this ring that is cut by the minimal-intensity ring of the second beam. Therefore, the beam in Figure 3k,l also has two light rings, as in the initial plane in Figure 3e,f.

Upon propagation into the Fresnel zone and the far field (rows 3 and 4 in Figure 3), the light rings of both BG beams almost do not overlap (rings in Figure 3m,o in the Fresnel zone and rings in Figure 3s,u in the far field). Therefore, after multiplication, these rings are suppressed, and other rings appear: those that are not seen in the initial plane. Thus, the diameter of the outer light ring of the product beam (Figure 3q,w) exceeds the ring diameters of both BG beams.

**Figure 4:**
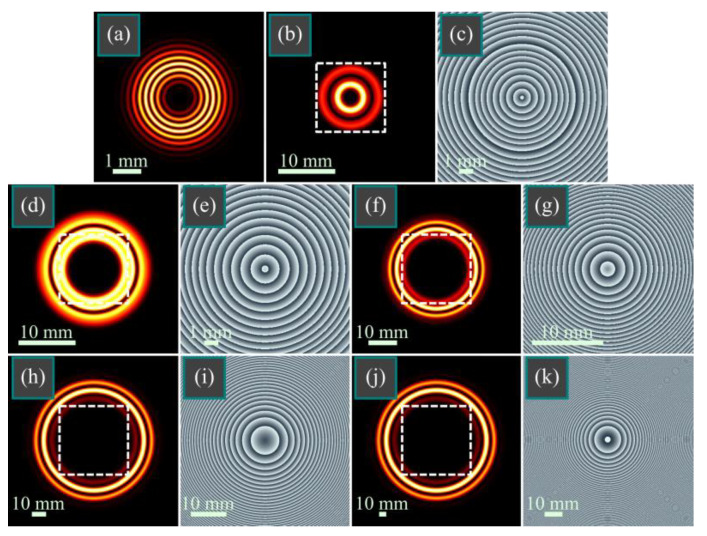
Intensity (**a**,**b**,**d**,**f**,**h**,**j**) and phase (**c**,**e**,**g**,**i**,**k**) distributions of the vortex-free power-function Bessel–Gaussian beam (17) in several transverse planes for the following computational parameters: wavelength λ = 532 nm, Gaussian beam waist radius *w*_0_ = 1 mm, beam order *m* = 3, scaling factor *a* = 15, propagation distances are *z* = 0 (**a**), *z* = *z*_0_/2 (**b**,**c**), *z* = *z*_0_ (**d**,**e**), *z* = 2*z*_0_ (**f**,**g**), *z* = 5*z*_0_ (**h**,**i**), and *z* = 10*z*_0_ (**j**,**k**). Dashed squares (**b**,**d**,**f**,**h**,**j**) denote the areas corresponding to shown phase distributions (**c**,**e**,**g**,**i**,**k**).

Figure 4 depicts the intensity and phase distributions of the vortex-free power-function BG beam (17) in several transverse planes.

As can be seen in Figure 4, the intensity distribution in the initial plane consists of multiple light rings (there are seven rings in Figure 4a). Upon propagation, only two rings remain; then, in the far field, the number of rings increases to three, which is consistent with the theory that predicts that there should be *m* rings in the far field. The phase distribution is not shown for the initial plane since it is zero, while in other planes, it is seen to be a rotationally symmetric, and the beam does not contain optical vortices.

Thus, in this section, the numerical simulation confirms our main theoretical ideas. First of all, the simulation shows that all the derived mathematical expressions are correct. Second, the hypothesis of the approximate shape-invariance of the modulated BG beams is confirmed when the scaling factor of the Bessel function is large enough. Third, the simulation confirms that the vortex-free power-function BG beam contains in the far field several light rings whose number equals to the beam order. In addition to confirming the theoretical predictions, the simulation demonstrates a feature of the product of two BG beams that was not predicted by the above theory. In contrast to the conventional BG beams that have one bright ring with many side lobes, the products of two BG beams contain two bright rings in the initial plane, in the Fresnel diffraction area, and in the far field since the bright ring of one BG beam in the product is being cut by an intensity at zero or at the minima of the other BG beam.

## 6. Conclusions

In this work, we obtained the following results. An integral transform (7) was derived that relates the standard BG beams, Equation (1), and the modified qBG beams, which are different from the conventional qBG beams, Equations (4) and (5). As can be seen from Equation (7), these beams can be treated as the continuous superposition of the standard BG beams, with the weight function equal to a fractional-order Bessel function. Square BG (BBG) beams (9) were proposed and studied, and their complex amplitude depends on the square of the Bessel function. We obtained the complex amplitude of the BBG beams in the Fresnel diffraction zone in the form of a series of products of three different Bessel functions (11). As a generalization of square BG beams, we also investigated double BG (dBG) beams (12), and their complex amplitudes are proportional to the product of two Bessel functions of different orders and of different scales. The complex amplitudes of such beams in the Fresnel diffraction zone were also represented in the form of a series of products of three different Bessel functions (13). We also considered modified BG beams, whose complex amplitudes are equal to a product of the Bessel function by the power function of the radial variable (16). This set of pBG beams is a subset of vortex-free BG beams. When such a beam of the *m*-th order propagates in free space, it becomes a superposition of a finite number of similar vortex-free power-function BG beams of the orders from 0 to *m*. Among the listed modifications of the BG beams, the double BG beams are the most general. They constitute a four-parametric beam family in which two parameters are integers (orders) and two parameters are complex-valued. If the complex parameters become real-valued, they define the beam scales. The square BG beams and the power-function BG beams are special cases of the double BG beams. When one order and one scale parameter are both equal to zero, the double BG beams reduce to the conventional BG beams from [2]. Thus, the family of the considered beams is significantly wider than the family of the standard BG beams and has two times more degrees-of-freedom. New varieties of the BG beams considered in this work will be useful for probing the atmosphere, in wireless communications, microparticle manipulation, and in quantum informatics for generating entangled pairs of photons. In micromechanics, these laser beams can be used for controlling the movements of microparticles along circular trajectories [31,32,33]. For instance, the one-dark-spot distribution of the square BG beams (from Figure 2) can be adopted for trapping a non-spherical metallic microscopic object and for rotating it around its center of mass, whereas the two-ring distributions of the double BG beams (from Figure 3) can be used for guiding metallic particles along circular paths since such particles, in contrast to dielectric ones, tend toward dark regions instead of bright regions. In addition, metallic particles tend toward an intensity minimum; thus, the squared BG beams can also allow the rotation of metallic particles (the intensity between the two bright rings in Figure 2g,k,o is nearly two times lower than the intensity on these rings). In optical data transmission, two-ring distributions can be used for redundant information encoding since it is more likely that medium-induced distortions will destroy one light ring than two.

## Data Availability

Not applicable.
